# The Use of Very-High-Resolution Aerial Imagery to Estimate the Structure and Distribution of the *Rhanterium epapposum* Community for Long-Term Monitoring in Desert Ecosystems

**DOI:** 10.3390/plants10050977

**Published:** 2021-05-13

**Authors:** Meshal M. Abdullah, Zahraa M. Al-Ali, Mansour T. Abdullah, Bader Al-Anzi

**Affiliations:** 1 Department of Ecology and Conservation Biology, Texas A&M University, College Station, TX 77843, USA; 2 Natural Environmental Systems and Technologies (NEST) Research Group, Ecolife Sciences Research and Consultation, Hawally 30002, Kuwait; zahraamma84@gmail.com (Z.M.A.-A.); kuwait79@gmail.com (M.T.A.); 3 Science Department, College of Basic Education, The Public Authority for Applied Education and Training, Kuwait City 12064, Kuwait; 4 Department of Environmental Technologies and Management, College of Life Sciences, Kuwait University, Kuwait City 13060, Kuwait

**Keywords:** unmanned aerial vehicles (UAVs), multispectral sensors, arid ecosystems, ecological monitoring and assessment, restoration, plant heights

## Abstract

The rapid assessment and monitoring of native desert plants are essential in restoration and revegetation projects to track the changes in vegetation patterns in terms of vegetation coverage and structure. This work investigated advanced vegetation monitoring methods utilizing UAVs and remote sensing techniques at the Al Abdali protected site in Kuwait. The study examined the effectiveness of using UAV techniques to assess the structure of desert plants. We specifically examined the use of very-high-resolution aerial imagery to estimate the vegetation structure of *Rhanterium epapposum* (perennial desert shrub), assess the vegetation cover density changes in desert plants after rainfall events, and investigate the relationship between the distribution of perennial shrub structure and vegetation cover density of annual plants. The images were classified using supervised classification techniques (the SVM method) to assess the changes in desert plants after extreme rainfall events. A digital terrain model (DTM) and a digital surface model (DSM) were also generated to estimate the maximum shrub heights. The classified imagery results show that a significant increase in vegetation coverage occurred in the annual plants after rainfall events. The results also show a reasonable correlation between the shrub heights estimated using UAVs and the ground-truth measurements (*R*^2^ = 0.66, *p* < 0.01). The shrub heights were higher in the high-cover-density plots, with coverage >30% and an average height of 77 cm. However, in the medium-cover-density (MD) plots, the coverage was <30%, and the average height was 52 cm. Our study suggests that utilizing UAVs can provide several advantages to critically support future ecological studies and revegetation and restoration programs in desert ecosystems.

## 1. Introduction

During the past few decades, considerable attention has been given to the development of improved revegetation and ecosystem restoration in dry areas. This comes in response to more rapid and widespread land degradation caused by climate change and anthropogenic activities [1,2,3]. Vegetation plays an essential role in combating desertification, enhancing human wellbeing, and reducing greenhouse gases through carbon sequestration [3,4,5]. However, revegetation in dryland areas is an onerous task owing to a variety of interacting factors, including scarcity of water resources [6], unpredictable drought [7,8], salt-affected soil [9], limited acclimatization of species to harsh weather [10], human population growth, poor land mismanagement, and inadequate policies for ecosystem management [11]. Moreover, drylands are considered a particular case compared with other biomes with regard to their vulnerability to deterioration even under minor disturbances, slow recovery, low resilience, and sparse desert vegetation [2,12,13]. Introducing a standardized monitoring protocol will support future strategic planning by providing rapid assessments of the current site condition. Whether we assess the success of a restoration plan or investigate vegetation changes, selecting the optimum standardized monitoring method remains a key step for evaluating the success or failure of ecological processes.

Perennial desert shrubs play a significant role in enhancing the desert ecosystem and the health of its surrounding biological activities [14]. Native shrubs are also critical in stabilizing the desert surface and regulating the rainfall distribution [15]. Within this context, the rapid assessment and monitoring of native desert plants are essential for (1) understanding the success and failure of restoration and revegetation projects [16]; (2) understanding the interaction between earth and atmosphere, such as the impacts of climate change, drought, and soil erosion [17]; (3) tracking the changes in vegetation patterns in terms of vegetation coverage, as well as water and nutrient stresses; and (4) detecting the changes in leaf color showing symptoms of any disease [18,19,20,21]. However, the efficacy and the success of the vegetation monitoring are predicated on the selection of appropriate ecological indicators and methods considering both spatial and temporal scales [22]. The traditional techniques for vegetation monitoring, such as using transect lines and quadrat methods, have several constraints, such as being time- and money-intensive; the need for extensive labor efforts (human resources) in terms of field measurements, sample collection, and analysis; and the difficulty in accessing some study areas [23].

To that end, advanced remote sensing (RS) technologies using satellite imagery have been widely used in ecological studies because they provide accurate information rapidly and continuously at a lower cost [24,25]. Moreover, this technology can be integrated with ancillary data in a geographic information system (GIS) to interpret the physical attributes associated with land degradation. Several studies have highlighted the advantages of remote sensing in long-term monitoring projects, focusing on different aspects, as some researchers used spectral measurements derived from numerous satellite sensors, such as vegetation indices (VIs), to evaluate vegetation coverage [26,27,28]. Other studies have focused on tracking the seasonal dynamics of vegetation [29], detecting vegetation and land changes [30], and assessing ecological revegetation and restoration projects [31,32,33,34,35,36].

In addition to the advantages of utilizing RS techniques, there are several challenges and limitations associated with the use of medium- and high-resolution satellite imagery, particularly with native desert vegetation [37,38,39]. These limitations include the difficulty in detecting and distinguishing different desert vegetation species due to the sparsely distributed small-sized shrubs [20], as well as the unavailability of appropriate satellite imagery on cloudy days [32]. However, the rapid development of specialized technologies, such as unmanned aerial vehicles (UAVs) installed with remote sensing systems and multispectral sensors, can minimize the limitations or deficiencies that are often experienced when working with satellite imagery. UAV-based remote sensing imagery offers vital advantages over satellite imagery because it provides very-high-resolution imagery (up to 2.0 cm) at a lower cost. Additionally, UAV images are on-demand and can fill the data gaps on cloudy days [32,40], while there is also the potential to install a variety of sensors onboard the UAV-based platform [41].

The monitoring of plant cover density and height is critical in ecological studies because these are among the essential ecological indicators for studying plant growth and the development of biomass carbon stocks [42]. Furthermore, investigating rapid estimation methods of plant height and coverage is important for the long-term monitoring and assessment of restoration and revegetation programs [43,44]. UAVs can provide essential information to detect plant height [45]. These data can provide direct information regarding plant development and productivity, as well as indirect information regarding soil health, nutrients, and soil–water interactions [46,47,48,49]. However, monitoring the growth of desert plants using UAVs remains a challenge due to the small vegetation structure of plant communities. Previous studies proposed many remote sensing methods for estimating vegetation height [36]. Unfortunately, most studies have used complicated and expensive methodologies, such as light detection and ranging (LIDAR) [34,50,51]. Other studies have utilized less complicated methods, such as digital aerial photography (DAP) and multispectral photogrammetric cameras, to estimate vegetation height in forests and agricultural areas [52,53].

Several studies focused on using UAV-based platforms in monitoring vegetation in different regions and ecosystems. The United States leads the countries where most UAV research has been conducted, followed by Canada, Australia, and China [54]. However, very few studies utilizing UAVs have been conducted in the Gulf Cooperation Council (GCC) region, especially studies that investigate shrublands [54]. Arid lands have unique characteristics regarding vegetation density and distribution, such as having sparse vegetation and small shrubs (<1.5 m height), making them more challenging to monitor. Thus, the main objective of this paper was to investigate advanced monitoring methods utilizing UAVs for rapid assessment of the structure and distribution of native desert shrubs in Kuwait, as well as to infer their relationship with native annual plants. Specifically, we (1) assessed the vegetation response to extreme rainfall events; (2) examined the use of very-high-resolution aerial imagery to estimate the vegetation structure of a perennial desert shrub (*Rhanterium epapposum* Oliv., Asteraceae) by integrating UAV-based multispectral remote sensing and GIS; and (3) investigated the relationship between the distribution of perennial shrub structure and vegetation cover density of annual plants using UAVs. The outcome of this work will support monitoring restoration and re-vegetation projects through assessing the growth of native perennial shrubs in arid landscapes.

## 2. Materials and Methods

### 2.1. Study Area

**Figure 1:**
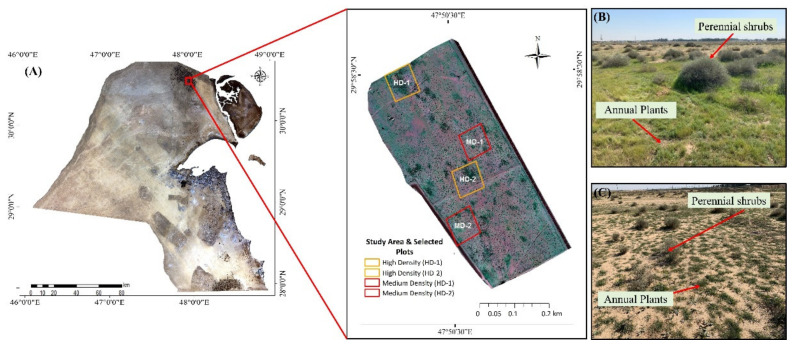
Al-Abdali study area: (**A**) map representing high-vegetation-cover-density plots (HD-1 and HD-2) and medium-vegetation-cover-density plots (MD-1 and MD-2); (**B**) example of high cover density, including perennial shrubs and annual plants; (**C**) example of medium cover density, including perennial shrubs and annual plants.

This work was conducted at the Al Abdali fence-protected site (total area of 15 acres), which is located in one of the largest agricultural areas in the northeastern part of Kuwait City (29°58′15.68″ N and 47°50′29.94″ E) (Figure 1A). Kuwait is characterized as an arid ecosystem with long, dry, and hot summers (average maximum temperature ranges from 42 to 46 °C), short warm winters (average minimum temperature ranges from 3 to 13 °C), and only occasional rainfall, i.e., an average of 112 mm annually [55,56,57]. The topography of the study area is generally flat and smooth with slight undulation. The area is protected by a 2-m-high fence consisting of naturally occurring native desert vegetation, which is dominated by *Rhanterium epapposum*, a perennial shrub, also known as the national flower of Kuwait. During the spring season, diverse tiny native annual plants (<30 cm height) are distributed throughout the area surrounding the *Rhanterium* shrubs (Figure 1B). Commonly occurring annual plants are represented by *Astragalus schimperi* Boiss., *Launaea nudicaulis* (L.) Hook.f., *Ifloga spicata* (Forssk.) Sch. Bip., *Lotus halophilus* Boiss. & Spruner, *Medicago laciniata* (L.) Mill., *Picris babylonica* Hand.-Mazz., *Plantago albicans* L., *Plantago ovata* Forssk., *Reichardia tingitana* (L.) Roth., *Senecio glaucus* subsp. Coronopifolius (Maire) Alexander, *Silene villosa* Forssk., and *Stipa capensis* Thunb.

### 2.2. Estimating Shrub Coverage before and after Rainfall Events Using UAVs

**Figure 2:**
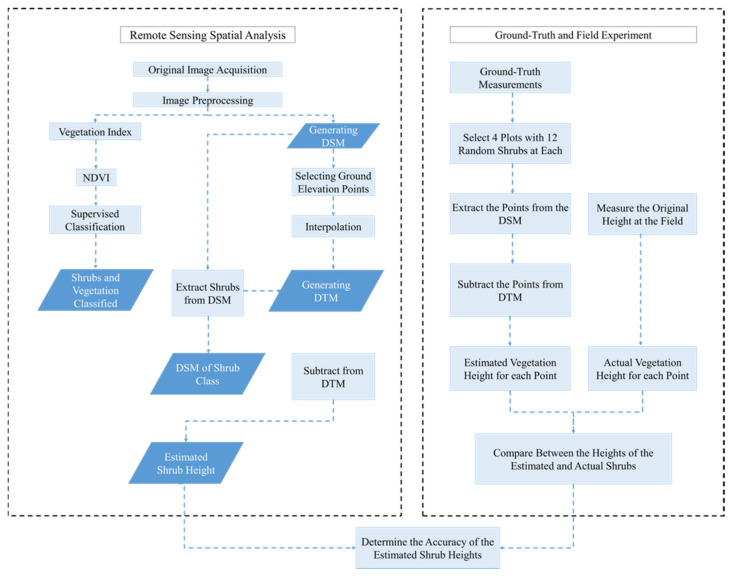
Chart showing the workflow methodology.

UAV multispectral imagery was collected in October and December of 2018 and January of 2019 to monitor the changes occurring in plant growth and development (including perennial shrubs and annual plants) before and after rainfall events. In addition, for the analysis of plant cover density and maximum height, the images were processed and analyzed using vegetation indices (VI) and image classification methods. The detailed methodology is discussed below (Figure 2).

### 2.3. Image Data Acquisition

**Figure 3:**
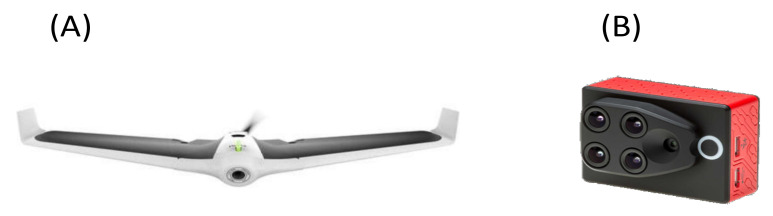
UAV platform, ready-to-fly kit: (**A**) Parrot Disco-Pro AG; (**B**) Sequoia multispectral sensor.

A Parrot Disco-Pro AG (ready-to-fly kit) fixed-wing UAV was used in this study (Figure 3A) accompanied by a Parrot Sequoia multispectral sensor capable of sensing multispectral bands that included green (550 BP 40), red (660 BP 40), red edge (735 BP 10), and near-infrared (790 BP 40) (Figure 3B) [58]. The UAV weighed 780 g without the Parrot Sequoia camera, whereas the takeoff weight was 940 g with the Parrot Sequoia and mount. The UAV size was 1150 × 580 × 120 mm (45 × 22 × 5 in), with a wingspan of 1150 mm (45 in). The Parrot Disco-Pro AG allowed covering 80 ha (200 ac) in a single flight at a flight altitude of 120 m (400 ft). The Parrot Sequoia multispectral bands analyzed the vitality of plants using vegetation indices, which mainly focus on capturing the amount of light they absorb and reflect, as well as RGB photos. The Parrot Sequoia multispectral sensor also contained a sunshine sensor, which recorded the sunlight intensity to perform radiometric calibration to ensure consistent data measurement. The ground resolution of the sensor was 11.3 cm/px at 120 m flight altitude [58].

### 2.4. Image Data Collection and Processing Using UAVs

#### 2.4.1. Image Data Collection

In this study, UAV images were collected with a spatial resolution of 7.2 cm/px at an 80 m flight altitude. A total of 4200 images were captured in one flight to cover the entire study area, including the RGB and multispectral bands. The UAV images were obtained before the rainfall event on 15 October 2018 and after the rainfall events on 23 December 2018 and 20 January 2019. An automatic flight plan was followed through the Pix4D capture mobile application (www.pix4d.com, accessed on 1 January 2018). The images were collected with an 80% overlap to account for corrupted images affected by the wind speed. High imagery overlap (80%) was selected to provide more details of the vegetation geometry and to minimize the vegetation height error [59,60,61]. Imagery products were georeferenced and radiometrically corrected and mosaicked using Pix4Dmapper software (version 4.3). Pix4Dmapper was also used to generate a digital service model (DSM), digital terrain model (DTM), and spectral bands. The processing steps for the Pix4Dmapper to create these layers were as follows: (1) initial processing, with keypoint extraction and matching, camera model optimization, and GPS geolocation; (2) point-cloud and mesh establishment, whereby tie points were created on the basis of the densified point cloud and a 3D textured mesh was generated; and (3) orthomosaic and index establishment, whereby the DSM, DTM, and orthomosaic and reflectance layers were created.

#### 2.4.2. Vegetation Indices (Vis)

Vegetation indices are widely used in monitoring/assessing vegetation coverage and distribution. In this study, the normalized difference vegetation index (NDVI) was used to distinguish between healthy vegetation and bare ground, as well as to identify the plant patterns and distributions within the study area [39]. NDVI can separate green vegetation from its background soil brightness and is defined as the difference between the near-infrared (NIR) and red (RED) bands normalized by the sum of those bands [62]. It is also considered the most widely used index for assessing vegetation dynamics at the local, regional, and global scales [63]. Our previous work showed consistent results in determining native desert vegetation in the State of Kuwait [12,55,64]. The following Equation was implemented to generate the NDVI layer:

$$\mathrm{NDVI}=\frac{\left(NIR-RED\right)}{\left(NIR+RED\right)}$$

#### 2.4.3. Image Classification

Supervised classification was utilized in this work to classify the images using ENVI (5.3) software. The generated NDVI was stacked with the remaining multispectral bands to help determine the different land-cover classes by selecting the training regions of interest (ROIs) [65]. Three classes of interest were defined: (1) perennial shrubs; (2) annual plants; and (3) bare ground. The NDVI was utilized to select the ROIs for the shrubs and annual plants to identify healthy green vegetation. The support vector machine (SVM) classifier method was selected to classify the stacked images. The SVM classifier is a binary machine-learning algorithm that classifies pixels by locating the optimal statistical boundaries between classes [66]. This classifier was selected in this work because it presented highly accurate results in detecting desert vegetation as well as distinguishing between perennial shrubs and annual plants; the methods are described in detail in previous studies [39,55,67].

### 2.5. Assessing Changes in Vegetation Coverage before and after Rainfall Events

According to the data obtained from the Kuwait Meteorological Center, Al Abdali Station, 17.9 mm of total rainfall occurred in October 2018 and 59.2 mm of total rainfall occurred in December 2018. The SVM-classified imagery was then compared to determine changes in perennial shrubs and annual plants after rainfall events. Changes in the NDVI values before and after rainfall events were also evaluated since native perennial shrubs may not show any significant changes in coverage but may increase the NDVI value, representing the health status of the plant. Changes in the NDVI value were estimated by extracting the shrub class from the NDVI layer for the three examined months using ArcGIS and the quick statistics tool in ENVI. Lastly, one-way analysis of variance (ANOVA) and the Tukey test were implemented to determine the significant changes in the NDVI values.

### 2.6. Estimating Maximum Shrub Heights Using UAV Images

A primary step in estimating maximum shrub height is to subtract the DSM from the DTM [68]. The DSM layer was generated on the basis of the densified 3D point cloud using Pix4Dmapper software, representing the height of all image objects, including shrubs, fences, buildings, rocks, and the ground. A digital terrain model (DTM) was generated to separate the vegetation height from the ground elevation. In this work, we examined two DTM layers using two different methods to determine the more accurate DTM method for estimating shrub height. This comparison was implemented to test the accuracy and resolution of the DTM generated by the UAV compared with the DTM layer generated by collecting ground points. The first DTM was generated from the UAV image using the ‘Triangulation’ method in the Pix4Dmapper software. This method is more practical and straightforward as it is generated from the UAV image using Pix4Dmapper software, where field data are not required. However, the second DTM layer was generated by collecting 30 ground elevation points in the field using a handheld Trimble with an ArcPad Global Positioning System with an accuracy ≤ ±30 cm. The points were interpolated using the ArcGIS interpolation tool to develop the DTM layer, which represents only the ground elevation beneath the vegetation canopies. Then, the perennial shrub class layer was extracted from the DSM to determine the surface height of the shrub class using GIS ArcMap. After that, the DSM layer representing the shrubs was subtracted from both DTMs to generate the final vegetation height and compare the accuracy of both DTMs in estimating the shrub heights.

### 2.7. Field Experimental Setup for Model Validation

**Figure 4:**
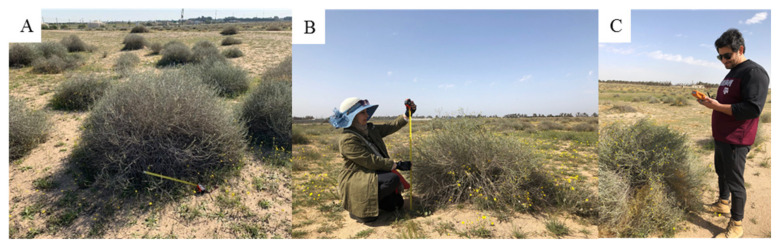
(**A**) Perennial shrubs (*Rhanterium epapposum*); (**B**) maximum shrub height was recorded using a measuring tape; (**C**) geographic localization using an accurate ArcPad GPS.

Given that this work focused on developing a rapid method to estimate vegetation cover density and maximum vegetation height using fixed-wing UAV-based remote sensing platforms, we also designed an experiment to quantitatively obtain the ground-truth heights of perennial shrubs in the field (Figure 2 and Figure 4). Field measurement was an important step to validate the accuracy and reliability of the estimated maximum plant heights using UAV imagery vs. actual measurements of the shrubs. In this study, the term plant height or maximum height refers to the length of the main stem from the ground surface to the maximum plant canopy height. In total, 70 perennial shrub sample points were selected randomly using the UAV image in ArcMap. The points were then transferred into ArcPad GPS to obtain ground-truth values for each point, and the maximum height of each selected perennial shrub in the field was recorded using a measuring tape. The results of the estimated shrub heights were then compared with the ground-truth points measured in the field. Regression analysis, which included the coefficient of determination (*R*^2^) and the root-mean-square error (RMSE), was implemented using JMP statistical software (version 13.0) to validate the results.

### 2.8. Relationship between Perennial Shrub Height and Annual Plant Cover Density

Arid lands have unique characteristics regarding vegetation distribution and height, having sparse vegetation and small shrubs (<1.5 m height). According to our monthly survey from September 2018 to March 2019, it was observed that the vegetation patches were distributed around the study area with a different vegetation cover density (including both perennial shrubs and annual plants). Some patches at the study site contained dense vegetation cover and others featured low cover. It was observed that dense-vegetation-cover patches were mainly associated with the high cover density of annual plants surrounding the perennial shrubs. To investigate the relationship between the perennial shrub heights and the cover density of annual plants surrounding the perennial shrub, we classified the study area into high-cover-density (HD) and medium-cover-density (MD) sites on the basis of the patchy pattern of the vegetation coverage. A total of four plots were selected in the study area: two plots represented high vegetation density (HD-1 and HD-2) and another two plots represented medium vegetation density (MD-1 and MD-2) (Figure 1). Plots with vegetation coverage > 30% were considered high-density vegetation cover (HD) and plots with vegetation coverage < 30% were considered medium-density vegetation cover (MD). The vegetation coverage was calculated using the NDVI layer in ArcGIS software. Twelve sample points of vegetation heights were selected in each plot. Then, regression analysis (including *R*^2^ and RMSE) was conducted to determine the relationship between the estimated and actual measured shrub heights within each plot. Next, the differences between the shrub heights within the selected plots were determined using one-way analysis of variance (ANOVA) and the Tukey test.

## 3. Results

### 3.1. Changes in Vegetation Coverage and Distribution after Extreme Rainfall Events

**Figure 5:**
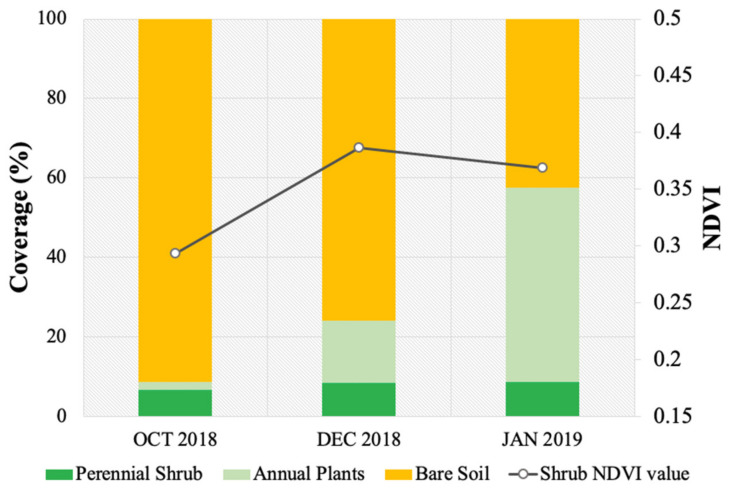
Changes in vegetation cover before rainfall events in October and after rainfall events in December and January.

In October 2018, the total vegetation coverage was 8.5%: 1.9% annual plants and 6.6% woody cylindrical stems branched from the base of the perennial shrubs (Figure 5). Following the high-intensity rainfall events recorded in November 2018 (261 mm), the total vegetation coverage significantly increased in December and January to reach 24% and 57%. The highest percentage cover increase was recorded for the annual plants, reaching 15% in December and 48% in January. However, the coverage of the perennial shrub (*Rhanterium shrubs*) slightly increased after the rainfall event to 8% in December and January. However, according to the ANOVA, the NDVI value showed a significant increase for the *Rhanterium* shrub mean (*p* < 0.001), which increased from 0.26 in October to 0.38 in December and 0.36 in January.

### 3.2. Vegetation Height Measurement Using UAV High-Resolution Imagery

**Figure 6:**
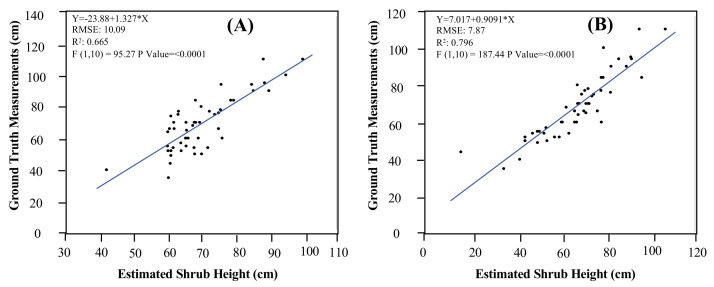
Correlations between UAV-estimated and ground-truth maximum shrub height: (**A**) correlation using the DTM generated from the ground-truth GPS points; (**B**) correlation using the DTM generated from Pix4D software.

The accuracy of the estimated shrub heights differed between the DTM generated from the UAV image and the DTM generated using the ground-truth GPS points. The results illustrated a reasonable correlation between the estimated shrub heights and the ground-truth measurements (*R*^2^ = 0.66, *p* < 0.01) using the DTM that was generated from the UAV image (Figure 6A). However, a stronger correlation was detected between the estimated shrub heights and the ground-truth measurements when using the DTM generated from the ground-truth points in the field (*R*^2^ = 0.79, *p* < 0.01) (Figure 6B). The RMSE also decreased from 10 cm to 7.9 cm when implementing the DTM generated from ground-truth points in the field.

**Figure 7:**
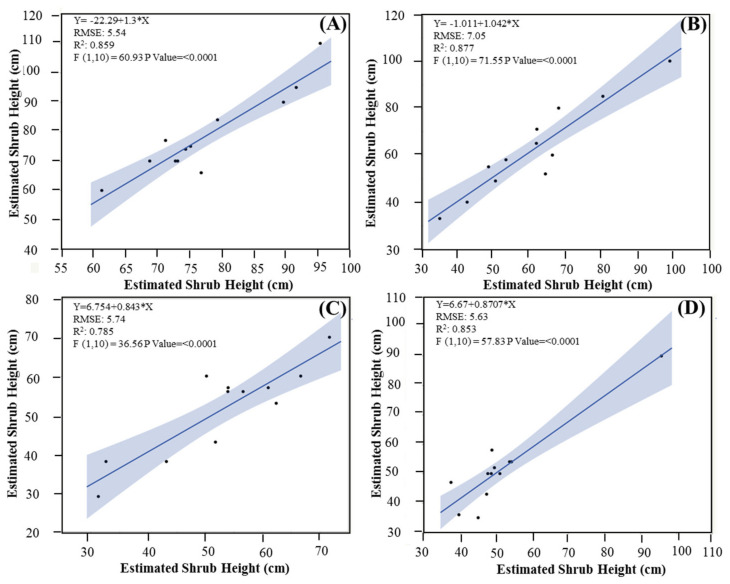
Correlations between the UAV-estimated and ground-truth (measured) shrub height for each plot: (**A**) high-density plot 1 (HD-1); (**B**) high-density plot 2 (HD-2); (**C**) medium-density plot 1 (MD-1); (**D**) medium-density plot 2 (MD-2).

Given that the DTM generated from the UAV showed promising results, it was utilized to estimate the shrub heights for the four selected plots: high-density plot 1 (HD-1), high-density plot 2 (HD-2), medium-density plot 1 (MD-1), and medium-density plot 2 (MD-2). These selected plots exhibited a strong correlation between the estimated shrub heights and the ground-truth measurements, with *R*^2^ = 0.87 for HD-1, 0.85 for HD-2, 0.78 for MD-1, and 0.85 for MD-2. Notably, all estimated heights were highly significantly correlated with the ground-truth heights of the shrubs (*p* < 0.0001) (Figure 7). The RMSE comparison between the UAV estimates and ground-truth measurements was >5.5 for all plots except HD-1, which showed a higher RMSE value of 7.7.

### 3.3. Relationship between Perennial Shrub Heights and Density of Annual Plant Coverage

**Figure 8:**
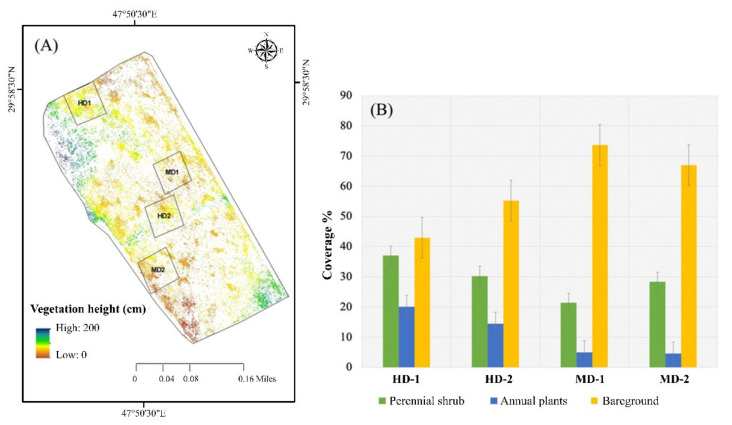
(**A**) Mapping the vegetation heights using the UAV platform; (**B**) percentage coverage of high- and medium-vegetation-density plots.

**Figure 9:**
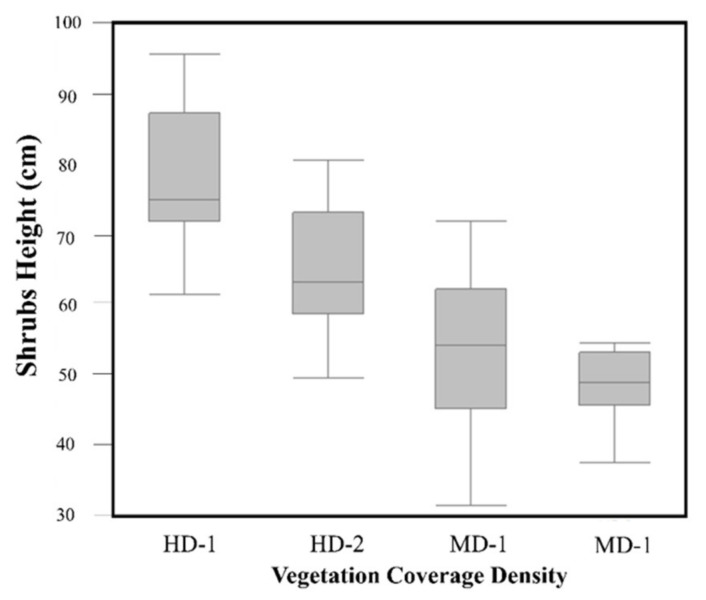
Shrub heights according to the plot density. Significant differences in the shrub heights were determined between the high- and low-density plots (*p* < 0.001).

The results indicated that the size of the shrubs differed within the study area. According to the classification map, shrub coverage was higher than annual plant coverage in all selected plots in October 2018. In the HD plots, the shrub coverage was 38% for HD-1 and 30% for HD-2, whereas, in the MD plots, it was 22% for MD-1 and 28% for MD-2. However, the annual plant coverage trend was significantly higher in the HD plots, which covered 20% of HD-1 and 15% of HD-2, whereas, in the MD plots, the annual plant coverage was <5 in both cases (Figure 8). It was also illustrated from the ANOVA results that the height of the shrubs was greater in the HD plots compared with the MD plots (*p* < 0.001) (Figure 9).

## 4. Discussion

### 4.1. Advantages and Disadvantages of Utilizing UAVs in Monitoring and Assessing Native Desert Shrubs

The results of this work show several advantages of utilizing very-high-resolution imagery with a UAV platform, which can critically support future ecological studies. It was found that UAVs can provide detailed information in estimating and monitoring the coverage and maximum heights of desert shrubs. One of the core advantages is that UAVs can help in understanding plant responses to extreme rainfall events, as well as distinguish between perennial shrubs and annual plants, which is one of the significant limitations of satellite imagery. Our results demonstrated that vegetation coverage differed between perennial shrubs and annual plants, as annual plants increased significantly after a rainfall event; however, perennial shrub coverage remained the same after rainfall events due to their life cycle and growth form. Changes were observed in annual plants during the growing season, with an increase in foliage coverage throughout the study area. Annual plant presence is only observed during the growing season (spring), with a high density of vegetation cover and maximum height reaching 30 cm [69]. The patterns of the annual plant distribution were irregular and patchily distributed throughout the study area, showing denser vegetation surrounding the *Rhanterium* shrubs. It was also observed that the NDVI value for the shrubs was generally low in October 2018, with an average value of 0.26; however, after the rainfall events, the NDVI value increased to 0.38 in December 2018 and 0.36 in January 2019 due to the growth and development of new leaves, indicating the health of the plants [70] as well as the blooming of the biocrust, which might also influence the NDVI value [71]. Therefore, it is important in vegetation assessment studies to consider the time when the image was captured due to the variation in vegetation cover and health.

Another vital advantage of utilizing UAVs is the ability to estimate desert shrub heights using 2D imagery with reliable accuracy, which is necessary for monitoring vegetation growth progress throughout the revegetation recovery process. Our results illustrated that the accuracy of the DTM influenced the efficiency of estimating maximum shrub height (Figure 6). It was found that the DTM generated from field GPS points showed a better correlation with vegetation shrub heights compared to the DTM produced from the UAV sensor. However, the DTM produced from the UAV sensor still provided reasonable results, while it is also associated with the most suitable practical utility since it is more time-efficient and requires less manpower to operate and process the data, particularly with regard to monitoring larger areas. Additionally, the estimated shrub heights using UAV imagery could significantly support the rapid assessment and monitoring of restoration and revegetation programs in arid landscapes. This methodology can also support decision-makers to track the progress of revegetation success by estimating the plant height and density within the restored area.

Even though UAVs can limit the disadvantages and predicaments of satellite imagery, especially when dealing with desert shrubs, they still have some limitations. A significant barrier is the inability of UAVs to encompass the massive size of open rangelands [72]. Therefore, it is essential to note that very-high-resolution imagery cannot replace medium- and high-resolution satellite imagery and aircraft-borne sensors in ecological studies. Both imagery types provide different information, and each type has its advantages and disadvantages. The selection of UAV imagery over satellite imagery is contingent on the scale, purpose, and requisite information for the study. In other words, UAVs are known to be useful for assessing vegetation communities, sizes, and heights within a miniature landscape; however, medium-resolution satellite imagery is useful for studying vegetation coverage within large landscapes in arid ecosystems.

The major challenge facing UAVs occurs when dealing with vast landscapes, as UAVs cannot cover large areas. We believe that we can still estimate whether plants are perennials or annuals using high- and medium-resolution satellite imagery by considering the NDVI values and locations where vegetation coverage appears during the year. For instance, sites where vegetation coverage is always present are more likely to represent perennials, as they survive for more than 2 years. During the off-season, when temperature increases and rainfall decreases, the perennial shrubs turn into dry woody plants with a low NDVI value, whereas they flourish again during the spring season with a significant rise in the NDVI value. However, vegetation types that disappear during the off-season (dry season) are most likely annual plants since their life cycle is short and highly dependent on rainfall events. Thus, satellite and UAV imagery can be integrated into restoration and revegetation programs, as satellite imagery can help estimate the life form of plants, while UAVs can be utilized to obtain ground-truth values for hotspot study plots.

Another critical challenge countered during this work is the number of overlapped captured images to cover the RGB and multispectral bands. In this work, captured images with 80% overlap (around 4222 images) were collected to cover the entire area and to account for images affected by the wind speed. High imagery overlap provides better-quality data with a decent level of detail and fewer errors in terms of the shrub heights. However, increasing the number of overlapping images will increase the flight time, leading to higher battery consumption. Furthermore, the processing time significantly increases with an increase in image overlap and accuracy (reducing pixel size), which then requires powerful computers to process the extensive data. Therefore, we believe that these aspects need to be considered in the project’s planning stage to avoid any complications with data processing during the implementation stage.

### 4.2. Factors Influencing the Size and Distribution of Desert Plants

The results demonstrated that the coverage of annual plants and the size of the perennial shrubs differed within the study site (Figure 8). This illustrates that some biotic and abiotic factors may influence the distribution and magnitude of native desert vegetation within a small rangeland area. Such differences in the plant heights were most likely related to the age of the shrubs, as larger shrubs may be older than smaller shrubs. On the other hand, the vegetation size and distribution may be significantly influenced by the density of the annual plants surrounding the shrubs, since it was found from the results that the shrubs were larger in the high-density vegetation plots surrounded by annual plants (Figure 8). The distribution of annual plants surrounding the shrubs could provide fundamental support to native desert shrubs by (1) decreasing the soil temperature; (2) decreasing evaporation directly from the soil surface, thus leading to greater availability of water in the soil for plant root uptake [73]; and (3) increasing the availability of organic matter and nutrients in the soil [74,75]. Therefore, it is probably paramount to conduct more detailed studies on the vegetation, water, soil, and atmosphere interactions utilizing UAVs. Such comprehensive studies will advance our understanding of the complexity of desert ecosystems.

## 5. Conclusions

This work indicated the success of using advanced UAV techniques in describing the status of native desert plants, mainly the height and cover distribution of perennials and annuals, as well as the reliable accuracy in estimating shrub heights. This work can also be implemented for rapid assessment and monitoring strategies to assess vegetation growth and development in restoration programs. We also believe that more critical information can be determined in future work, which can help researchers develop a quantitative characterization of natural systems, including plant–soil–water–atmosphere interactions for desert ecosystems. More detailed studies on the interactions among these ecological components will improve our understanding of the complexity of the ecological process, leading to better management of the scarce resources in arid regions. Lastly, we believe that further studies are required to prove the feasibility of utilizing UAVs to monitor natural arid ecosystems in different regions, which will stimulate the development of new methods and the production of a set of ecological indicators and monitoring frameworks.

## Data Availability

The data presented in this study are available on request from the corresponding author. The data are not publicly available due to the restrictions of EcoLife Sciences Research and Consultation Company since their equipment was used to collect the data for this study.
